# The Heterogeneous Interplay Between Metabolism and Mitochondrial Activity in Colorectal Cancer

**DOI:** 10.3390/jpm15120571

**Published:** 2025-11-28

**Authors:** Christophe Desterke, Yuanji Fu, Jorge Mata-Garrido, Ahmed Hamaï, Yunhua Chang

**Affiliations:** 1 Faculty of Medicine, University Paris Saclay, Institut National de la Santé et de la Recherche Médicale (INSERM) Unit UMRS-1310, 94800 Villejuif, France; 2 Institut Necker Enfants Malades, Université Paris Cité, Institut National de la Santé et de la Recherche Médicale (INSERM), Centre National de la Recherche Scientifique (CNRS), F-75015 Paris, France; yuanji.fu@inserm.fr (Y.F.); jmatag@unican.es (J.M.-G.); ahmed.hamai@inserm.fr (A.H.)

**Keywords:** colorectal cancer, mitoscore, keggmetascore, metabolism, mitochondria, transcriptome, R-packages, single-cell RNA-sequencing

## Abstract

**Background**: Colorectal cancer is a multifactorial malignancy implicating a wide variety of risk factors, such as genetic, environmental, nutritional, and lifestyle factors, leading to a certain heterogeneity in the development of the disease. Colorectal cancer is generally classified in terms of a Warburg metabolic phenotype, characterized by an excess of glycolytic axes as compared to oxidative phosphorylation. It is therefore important to better characterize the metabolic heterogeneity of these tumors in relation to their mitochondrial activity. **Materials and Methods**: Two R-packages (keggmetascore and mitoscore) were developed to explore metabolism, based on KEGG metabolism pathways, and mitochondrial activities, based on mitocarta V3 annotations, for the investigation of diverse transcriptomics data such as bulk or single cell experiments at the single-sample level. **Results**: Using the two R-packages, we functionally confirmed both regulation of metabolism and mitochondrial activities in LOVO cells after stimulation with metformin. At the single-cell level, in single-cell RNA-sequencing of colorectal tumors, we conjointly observed an activation of metabolism and mitochondrial activities in tumor cells from MSI-high tumors, in contrast to a conjoint repression of metabolism and mitochondrial activity in tumor cells from POLE-mutated tumors. These two types of tumors have distinct responses to immune checkpoint blockade therapy. At the bulk transcriptome level, colorectal tumors present less metabolism/mitochondria activities as compared to normal tissues. Multi-modal integration by co-expression network analysis showed that metabolism/mitochondrial activities are associated with a consensus molecular subtype (CMS) classification of colorectal cancer. Regarding KRAS, BRAF, and TP53 driver gene mutation status, strong repression of metabolism pathways was observed, mainly associated with fewer intra-mitochondrial membrane interactions in tumors harboring a BRAF-V600E mutation. Machine learning using Elastic-net allowed us to build a mixed metabolism/mitochondrial activity score, which was found to be increased in the CMS1-MSI subtype and metastatic samples and to be an independent parameter predictive of BRAF-V600E mutation status in colorectal cancer. **Conclusions**: These findings underscore the pivotal role of mitochondrial metabolism in colorectal cancer subtyping and highlight its value as a predictive biomarker for personalized therapeutic strategies.

## 1. Introduction

Colorectal cancer (CRC) is a major global health concern, ranking as the third most frequent malignancy and the second leading cause of cancer-related mortality worldwide [1]. Due to a wide variety of risk factors, such as genetic, environmental, nutritional, and lifestyle aspects, CRC presents significant heterogeneity in its development. Its incidence is projected to increase by sixty percent by 2035 [2].

At the cellular level, energy metabolism is crucial, with mitochondria serving as metabolic and signaling hubs that interconnect cellular metabolism and signaling pathways [3]. Mitochondria support respiration through the tricarboxylic acid (TCA) cycle and fatty acid beta-oxidation, which generate electron donors to fuel the electron transport chain (ETC) and ATP synthase for oxidative phosphorylation (OXPHOS) [4]. However, during cancer development, metabolism is often deregulated. Almost one century ago, Otto Warburg initiated research on mitochondria alterations in cancer and proposed a model to explain the differences in energy metabolism between normal and cancer cells [5,6]. CRC is classically categorized by the Warburg metabolic phenotype, defined by an excess reliance on glycolysis over mitochondrial OXPHOS for energy production [7,8]. This dichotomous view, focusing only on the switch between glycolysis and OXPHOS, may be overly simplistic and fails to accurately represent the metabolic heterogeneity inherent in colorectal cancer.

Furthermore, specific cancer-associated mutations, such as those found in nuclear-encoded mitochondrial enzymes like fumarate hydratase (FH) [9], succinate dehydrogenase (SDH) [9], and isocitrate dehydrogenase (IDH) [10], have been reported, leading to the definition of a distinct metabolic phenotype termed the “truncated TCA (tricarboxylic acid) cycle” [11]. Better characterization of this metabolic heterogeneity in relation to mitochondrial activity is therefore highly relevant, so understanding the deeper inter-connections between metabolic pathways and mitochondrial activities could be critical for precision oncology.

This complexity is magnified by the observation that different molecular subtypes of CRC exhibit distinct responses to therapy, notably immune checkpoint blockade (ICI) therapy (e.g., using Pembrolizumab). For example, studies have shown that patients with Microsatellite Instability-High (MSI-high) or MMR-deficient CRC may benefit from ICI [12]. Exploring metabolic and mitochondrial signatures in these subgroups is essential, as the opposite metabolic activity between MSI-high tumors and POLE-mutated tumors could provide a new angle to identify molecular targets and explain differences in ICI response. In contrast, tumors from the POLE-mutant/TMB-UH group displayed high levels of nsSNV-derived neoantigen burden and recurrent antigen presentation abnormalities of neo-antigen depletion, showing a relatively quiescent immunophenotype overall [13].

To enable the quantitative and multi-scale investigation of metabolism and mitochondrial activity in CRC transcriptomics data, we developed two specialized R-packages. The first, “keggmetascore”, is dedicated to profoundly characterizing metabolism based on 41 filtered vectors derived from KEGG metabolism pathways [14]. The second, “mitoscore”, utilizes Mitocarta V3 annotations [15], which form a catalogue of 1136 human genes assigned to 149 hierarchical mitochondrial pathways; this catalogue is dedicated to accurately examining and evaluating mitochondria activities in transcriptomics data at a single-sample level.

In this study, we apply these two packages to various public transcriptomics datasets to accurately investigate the metabolism and mitochondrial status of colorectal cancer at both bulk transcriptome and single-cell RNA-sequencing levels, aiming to link functional heterogeneity to molecular classifications, prognostic factors, and therapeutic implications.

## 2. Materials and Methods

### 2.1. Data Acquisition from Public Datasets

#### 2.1.1. Bulk Transcriptome Data on Lovo Cells Stimulated by Metformin (GSE67342)

Metformin, a first-line drug used to treat type 2 diabetes, has been shown to have anticancer effects against a variety of malignancies, including colorectal cancer. Metformin suppressed the proliferation of LoVo cells and induced a time-dependent metabolic and transcriptional alteration [16].

During this study, human-derived LoVo cells (ATCC CCL-229) were treated with metformin (10 mM) for 8 and 24 h. The control and treated cells were harvested for RNA extraction and hybridization on Affymetrix microarrays. [HG-U133_Plus_2] Affymetrix Human Genome U133 Plus 2.0 Array. After microarray processing, the Affymetrix^®^ GeneChip^®^ analysis software MAS version 5.0 was used to normalize data with Affymetrix default analysis settings and global scaling.

#### 2.1.2. Single Cell RNA-Sequencing Performed on Colorectal Tumors (GSE222300)

During this study, colorectal tumors and rectal adjacent tissue of patients under therapy anti-PD1 (pembrolizumab) [13] were processed to perform single-cell RNA-sequencing experiments. More precisely, these three samples were sample GSM6919588, corresponding to a Microsatellite Instability-High/Tumor indel burden-high (MSI-high/TIB-high) rectal tumor for a patient who achieved complete response; sample GSM6919589, normal rectum tissue; and sample GSM6919590, corresponding to a POLE-mutant/TMB-UH (Tumor mutational burden—ultra high) sigmoid tumor for a patient with progressive disease. On the total mRNA of the samples, single-cell gel beads in emulsion (GEMs) were generated on a GemCode Single Cell Instrument (10× Genomics, Pleasanton, CA, United States). Single-cell RNA-seq cDNA libraries were prepared using Chromium™ Single Cell 3′ V3.1 Reagent Kits (10× Genomics, Pleasanton, California, United States) according to manufacturer protocol. RNA libraries were generated by 10× Genomics Single Cell 3′ V3.1 Library Gel Bead Kit (10× Genomics, Pleasanton, California, United States) and prepared for sequencing using standard Illumina protocols. Basecalls were performed using CASAVA version 1.8. Cellranger (v3.1.0) was utilized to calculate the feature-barcode matrices. Cells containing over 200 expressed genes and a mitochondrial UMI rate below 40% passed the cell quality filtering process, and mitochondria genes were removed from the expression table. The Seurat package was used. Raw data were downloaded from the following web address: https://www.ncbi.nlm.nih.gov/geo/query/acc.cgi?acc=GSE222300 (accessed on 7 April 2025).

#### 2.1.3. Bulk Transcriptome of Primary Colorectal Tumors from Stage II (GSE44076)

This transcriptome dataset comprised 246 samples. Gene expression profiles were quantified for paired normal adjacent mucosa and tumor samples (primary colorectal tumors from stages IIa and IIb) from 98 individuals and 50 healthy colon mucosae (Table 1). Samples were processed through Affymetrix Human Genome U219 Array technology, and data were normalized with the Robust Multi-array Average (RMA) algorithm, which was used for data normalization through R and Bioconductor [17]. Normalized data were annotated via the corresponding technological platform GPL13667 [HG-U219] Affymetrix Human Genome U219 Array.

#### 2.1.4. Multi-Center Bulk Transcriptome of Primary Colorectal Tumors with Prognosis Meta-Data (GSE103479)

In this study, the colorectal cancer cohort was assembled from 363 stage II and III patients from 4 European Centers (Barcelona, Dublin, Florence, Aberdeen). Within this cohort, samples from 194 patients with 50% or more tumor content, as assessed by Hematoxylin and eosin (H&E) staining, were taken for RNA and DNA extraction. High-quality transcriptomics data were obtained for 156 samples using the Almac Xcel array (Almac Diagnostics, Craigavion, UK) [18]. Transcriptome data were processed by RMA normalization (Bioconductor R makecdfenv and affy packages), associated with batch correction by the ComBat algorithm (R sva package) [19]. A total of 137 processed samples matched those from the clinical meta-database (Table 2).

### 2.2. Software Development

#### 2.2.1. Development of the “Keggmetascore” R-Package

Development of the “keggmetascore” R-package was performed in R software environment version 4.2.2 and Rstudio version 2024.12.1. Starting from the human KEGG database [20], gene set signatures of metabolism were filtered to build data input as a list comprising 41 vectors of human gene symbols. With these 41 gene sets, the “keggmetascore” R-package allowed us to perform single sample gene set enrichment analysis with GSVA algorithm implementation [21]. Differential gene set enrichment analysis was also possible with the implementation of the LIMMA algorithm [22]. Finally, the “keggmetscore” R-package allowed us to perform graphical representations of gene set enrichments such as heatmap and volcanoplot. The “keggmetascore” R-package (version 1.0.1) was deposited in github at the address https://github.com/cdesterke/keggmetascore (accessed on 22 April 2025) and could be installed with the devtools command-line.

#### 2.2.2. Development of the “Mitoscore” R-Package

Development of the “mitoscore” R-package was performed in R software environment version 4.2.2 and Rstudio version 2024.12.1. Starting from mitocarta database version V3 [8], distinct lists of gene sets were built according to the three levels of annotations available in the original database. In this package, single sample gene set enrichment analyses were implemented though the GSVA algorithm [21]. This package also allows for graphical representation of mitochondria computed scores as a heatmap. The “mitoscore” R-package (version 1.0.0) was deposited in github at the address https://github.com/cdesterke/mitoscore (accessed on 22 April 2025) and could be installed with the devtools command-line.

### 2.3. Analyses

#### 2.3.1. Single Cell RNA-Sequencing Analyses

Bioinformatics analyses were performed in R software environment version 4.2.2. The three sc-RNAseq samples from dataset GSE222300 were pre-processed by filtering cells detected with fewer than one hundred expressed genes and by filtering features expressed in fewer than ten cells per sample. The three experiments were merged into a Seurat 5 single-cell object [23,24], and normalization was performed based on the definition of common anchor genes between samples. Dimension reduction was performed by principal component analysis and secondly by UMAP [25]; cell clustering was performed via a graph-based clustering approach by searching for neighbors in the fifteen first spatial dimensions [26]. Expression data were extracted from the Seurat object as count matrix, and metabolism/mitochondria scores were quantified with “keggmetascore” and “mitoscore” R-packages by applying the single-sample GSEA algorithm [21]. Results were integrated to the Seurat object as new data assays, as is described in the jupyter notebook dedicated to mitoscore quantification, linked here: https://github.com/cdesterke/CRC_metamito/blob/main/mitochondria.ipynb (accessed on 24 April 2025). Activity scores were graphically visualized as a dotplot, violinplot, and featureplot with Seurat functions.

#### 2.3.2. Colorectal Cancer Consensus via Molecular Subtype Classification

The CMScaller R-package version 0.99.2 was used to predict the consensus of molecular subtypes of colorectal cancer, as follows: CMS1: immune; CMS2: canonical; CMS3: metabolic signals; and CMS4: mesenchymal/stromal signal) [27].

#### 2.3.3. Weighted Gene Co-Expression Network Analysis

Metabolism and mitochondria scores quantified on the GSE103479 colorectal tumor bulk transcriptome were integrated in a common matrix to perform weighed gene co-expression network analysis with the WGCNA algorithm [28,29], package version 1.73. Soft-power was tuned as the first step to compute the optimal adjacency matrix and module identification for the metabolism/mitochondria activity score matrix in colorectal tumors. Phenotype–trait correlations were performed for the five identified gene set modules. For each significant module, scores/module connections were collected to build sub-networks with the Cytoscape standalone application, version 3.9.1 [30].

#### 2.3.4. Machine Learning by Elastic-Net to Predict BRAF-V600E Mutation Status with Metabolism/Mitochondria Activities

Metabolism and mitochondria scores quantified on the GSE103479 colorectal tumor bulk transcriptome were integrated in a common matrix and combined with the BRAF-V600E mutation status metadata as the binary outcome variable. After splitting the data into training and validation sets (0.7/0.3 ratio), the ElasticNet model (binary outcome: BRAF-V600E mutation status) was tuned for alpha and lambda parameters using the caret R package (version 7.0-1) [31]. The final Elastic-net model was fitted using the best alpha parameter (alpha = 0.1) with the glmnet R package (version 4.1-8) [32].

#### 2.3.5. Mixed Metabolism/Mitochondria Activity Scores

Coefficients for predictive negative scores (BRAF-V600E outcome) identified by Elasticnet were extracted to compute a mixed metabolism/mitochondria activity score according to their activity quantification in tumors, as follows:

MM_score = Σ coef Elastic Net ×Activity mitochondria/metabolism.

Optimal threshold cutpoint for MM_score was determined with cutpointr R-package version 1.2.0 [33].

#### 2.3.6. Survival Analyses

Univariate survival analysis was performed on overall survival time event data of transcriptome dataset GSE103479. The survival model was set with the survival R-package version 3.8-3 according to metabolism/mitochondria score group stratification [34], and a Kaplan–Meier plot was drawn with the survminer R-package, version 0.5.0.

#### 2.3.7. Multivariable Logistic Analysis

A multi-variable logistic regression model was built on BRAF-V600E mutation status as an outcome with generalized linear model function and binomial logit family [35]. This model included Duke staging, collecting center, and metastasis stage as the covariable metabolism/mitochondria activity score. A nomogram of the model was drawn with regplot R-package version 1.1.

### 2.4. Process of Metabolism and Mitochondria Multi-Modal Integration in Transcriptome Data of Colorectal Tumor Cells

**Figure 1:**
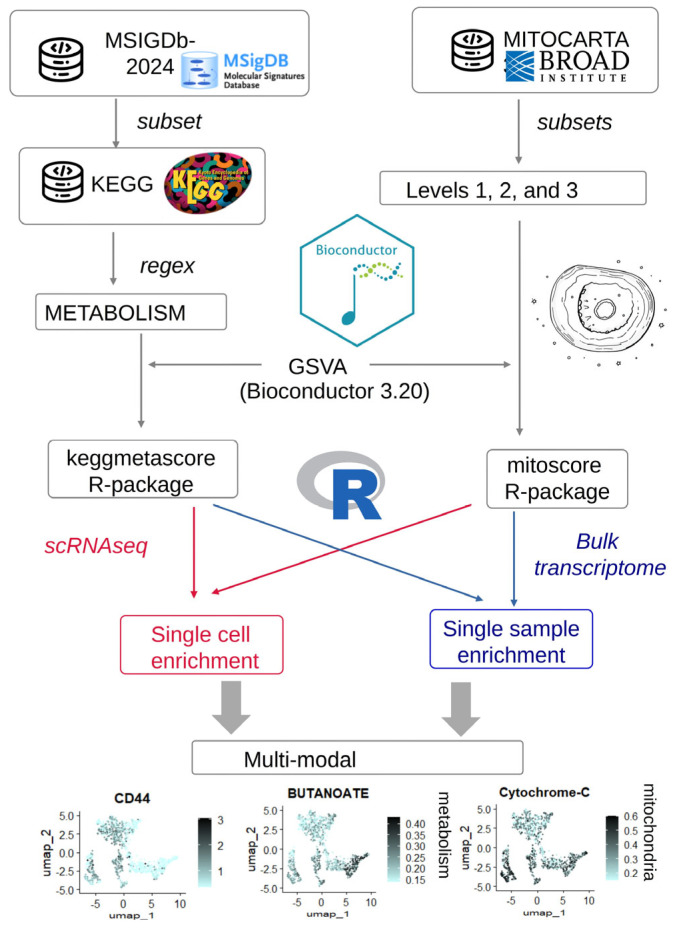
Workflow of bioinformatics strategy for the development of integrated mitochondria/metabolism scoring in transcriptome data. Starting from different databases, mitochondria and metabolism gene sets were built to design respective “mitoscore” and “keggmetascore” R-packages. Cell or sample transcriptome enrichment could be integrated for downstream analyses. Legends for arrows: blue arrows concerned process of bulk experiments, and red arrows concerned process of single-cell experiments.

The initial observation by Warburg that some tumor cells perform glycolysis at a high rate even in the presence of oxygen prompted him to formulate the hypothesis that mitochondria are not functional in tumors [5,6]. Mitochondria are double-membrane organelles containing a circular and double-strand genome. Their homeostasis is assured by active turnover depending on their biogenesis and their degradation, with dynamic changes in their size and morphology. Mitochondria and metabolism multi-modal integration in transcriptome data from tumor samples could help us understand the heterogeneity of this pathology according to the prognosis of the patient. For this reason, based on referent databases, KEGG from metabolism and mitocarta-V3 for mitochondria, respective “keggmetascore” and “mitoscore” R-packages were developed based on single-sample gene set enrichment analysis computation with expression data (Figure 1). For a demonstration of the utility of these packages, during this work, quantification was applied to the study of colorectal cancer transcriptomics data at the single-cell and bulk levels (Workflow, Figure 1).

## 3. Results

### 3.1. Metformin Conjointly Regulated Metabolism and Mitochondria After 24 h in LOVO Cells

**Figure 2:**
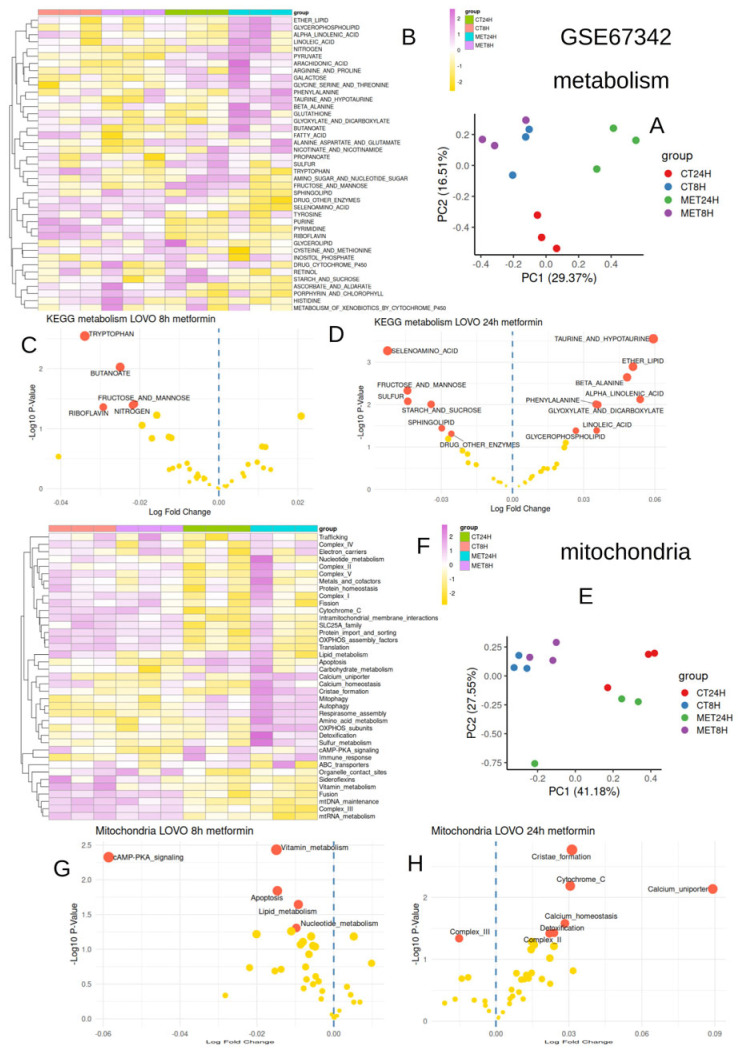
Early and late effects of metformin via in vitro stimulation on Lovo cells: bulk transcriptome GSE67342. (**A**): Principal component analysis performed on metabolism enrichment performed with “keggmetascore” R-package (CT: control, MET: metformin, 8H: early 8 h, 24H: late 24 h); (**B**): heatmap of single-sample gene set enrichment performed with “keggmetascore” R-package (metabolism); (**C**): volcanoplot of differential metabolism scores between metformin and control conditions at 8 h (early effect); (**D**): volcanoplot of differential metabolism scores between metformin and control conditions at 24 h (late effect); (**E**): principal component analysis performed on mitochondria enrichment performed with “mitoscore” R-package (CT: control, MET: metformin, 8H: early 8 h, 24H: late 24 h), (**F**): heatmap of single-sample gene set enrichment performed with “mitoscore” R-package (mitochondria); (**G**): volcanoplot of differential mitochondria scores between metformin and control conditions at 8 h (early effect); and (**H**): volcanoplot of differential mitochondria scores between metformin and control conditions at 24 h (late effect). In subfigures C, D, G, and H, red bullets refer to significantly regulated gene sets, and yellow bullets refer to non-significantly regulated gene sets.

A meta-analysis of observational studies indicates that metformin treatment lowers the risk of colon cancer in type 2 diabetes patients [36]. Metformin is also a poisoner of mitochondria by impairing the function of complex I, leading to increased aerobic glycolysis as compensation. The suppression of complex I prevents NADH oxidation, which results in the requirement for cytosolic NADH to be oxidized by converting pyruvate to lactate [37]. Metabolic and mitochondrial quantifications were performed, respectively, with keggmetascore and mitoscore R-packages on the transcriptome of LOVO cells stimulated in vitro with metformin during 8 and 24 h (dataset GSE67342, Figure 2). Principal component analysis performed on metabolism quantification showed a larger effect of metformin at 24 h than at 8 h (Figure 2A,B). At 8 h of stimulation, some metabolism pathways were found to be repressed, such as Tryptophan, Butanoate, Fructose/mannose, riboflavin, and nitrogen (Figure 2C). At 24 h, more metabolic pathways were found to be significantly regulated (Figure 2D). Concerning mitochondria functionalities, a larger regulation effect was still observed at 24 h of stimulation (Figure 2E,F). Few gene sets were found to be significantly repressed at 8 h of metformin stimulation, principally concerning cAMP-PKA signaling (Figure 2G). By contrast, at 24 h, metformin stimulation was found to have upregulated mitochondrial activity through cristae formation, cytochrome C, calcium uniporter and homeostasis, detoxification, and complex II (Figure 2H).

### 3.2. Metabolic and Mitochondrial Heterogeneity at Single-Cell Level in Colorectal Tumor Cells

**Figure 3:**
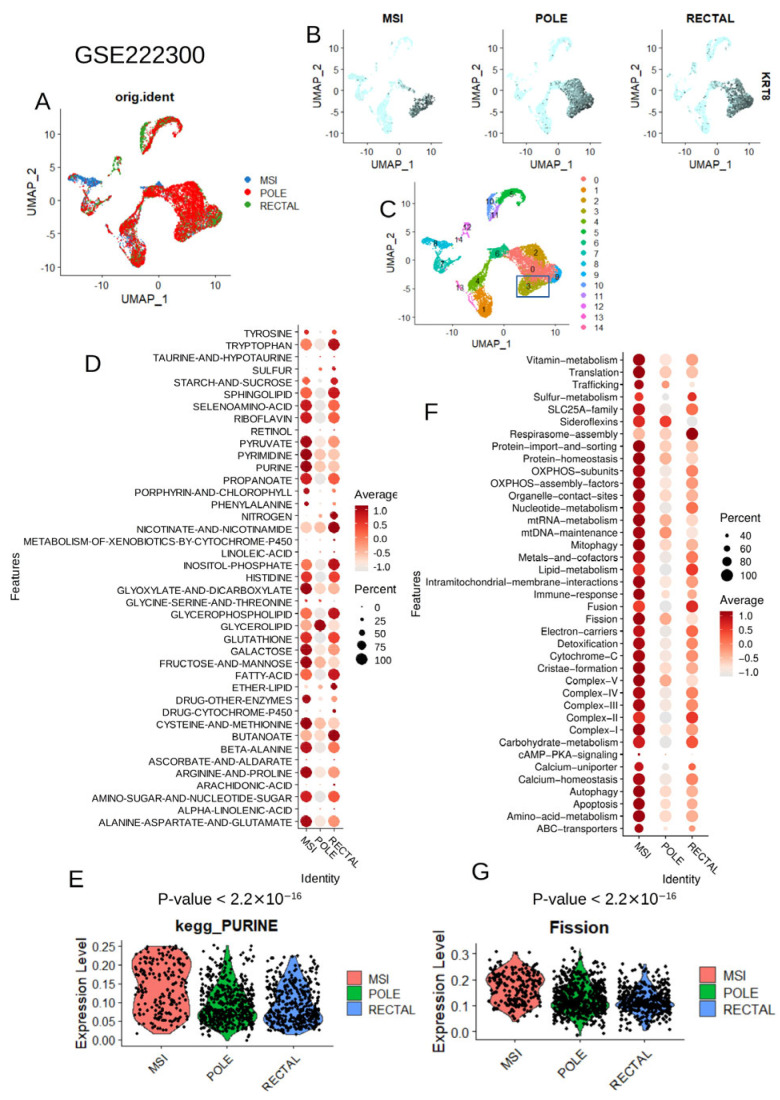
Heterogeneity of mitochondria and metabolism activation in tumor cells from colorectal cancer: single-cell transcriptome GSE222300, (**A**): UMAP dimension reduction in scRNAseq from one rectal tumor (RECTAL), one MSI colorectal tumor (MSI), and one POLE-mutated colorectal tumor (POLE); (**B**): featureplot of keratin 8 (KRT8) tumor cell marker stratified on sample origin, colors for expression level: low (cyan blue), high (dark grey); (**C**): UMAP dimension reduction focused on cluster 3 of tumor cells (blue box selection); (**D**): dotplot of single-cell metabolism enrichment (“keggmetascore” R-package) performed on cluster number “3” (tumor cells) and stratified on sample origin; (**E**): violinplot of purine metabolism enrichment stratified on sample origins (Fisher ANOVA *p*-value); (**F**): dotplot of single-cell mitochondria enrichment (“mitoscore” R-package) performed on cluster number “3” (tumor cells) and stratified on sample origin; and (**G**): violinplot of mitochondria fission enrichment performed on cluster number “3” and stratified on sample origins (Fisher ANOVA *p*-value).

Metabolism scoring (“keggmetascore” R-package) and mitochondria scoring (“mitoscore” R-package) were applied to the single-cell RNA sequencing in colorectal tumors from dataset GSE222300. A single-cell Seurat object was built with three distinct samples: normal tissue adjacent to rectum, rectal cancer complete response to immune-therapy (CR), MSI-high (Microsatellite Instability)/TIB-high (tumor indel burden), and sigmoid cancer, progression disease under immune therapy (PD), and POLE-mutant/TMB-UH (tumor mutation burden ultra-high > 100) (Figure 3A). MSI-high/TIB-high tumors exhibited a higher infiltration rate of activated immune cells, elevated immune checkpoint targets, and greater interaction between PD-1 and PD-L1, with increased immunogenicity mostly attributable to an abundance of indel-derived neoantigens. The keratin-8 epithelial marker was found to be expressed in right bottom cells from the UMAP in the three distinct samples (Figure 3B). After clustering, this right bottom part of the UMAP was stratified in cluster number 3 (Figure 3C). Focused on this cluster number “3”, cells from the three samples were found to express some epithelial/tumor markers at distinct levels, with markers including EPCAM, CD44, KRT8, ITGA6, and CLDN4 (Supplemental Figure S1). Quantification of metabolism and mitochondria activities were respectively quantified by “keggmetascore” and “mitoscore” R-packages in cells from the cluster number “3”. As a benchmark example, for the single-cell process for analyzing the “mitoscore” (level 2 for 39 mitochondrial gene sets) in 1156 tumor cells (cluster number “3”) from scRNA sequencing, the algorithm took eight seconds in a Google Colab session (equivalent to 12 GigaByte of Random Access Memory). The jupyter notebook for this data processing is available at the following address: https://github.com/cdesterke/CRC_metamito/blob/main/mitochondria.ipynb (accessed on 23 April 2025). Interestingly, after single-cell RNA-sequencing quantification, it was found that metabolism had a tendency to increase and mitochondria activity had a tendency to increase in the MSI-high tumor as compared to normal rectal mucosa. It concerned distinct metabolism pathways such as pyruvate, purine and pyrimidine, fructose and mannose, cysteine and methionine, glyoxylate and dicarboxylate, and arginine and proline (Figure 3D,E). By contrast, a reduction in metabolism was observed in POLE sigmoid tumor cells as compared to normal rectal mucosa (Figure 3D). Concerning mitochondria activity quantification, a major increase was quantified in cells from the MSI rectal tumor as compared to normal rectal mucosa (Figure 3F,G), and a major reduction in cells of the sigmoid POLE tumor was observed (Figure 3F).

### 3.3. Colorectal Tumors Harbored Major Repression of Their Metabolism/Mitochondria Activity as Compared to Normal Tissues

**Figure 4:**
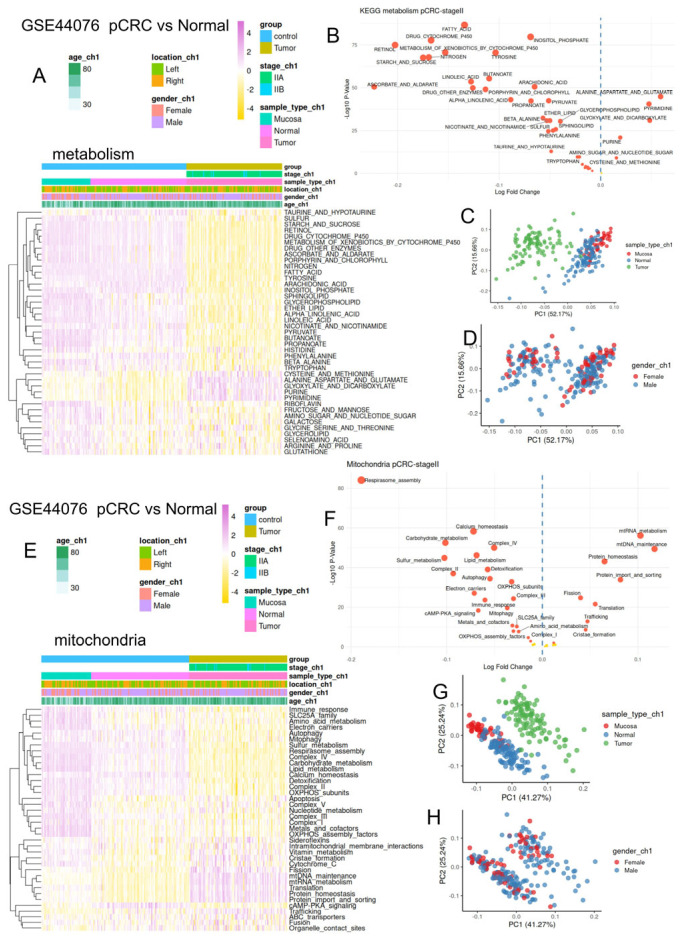
Co-repression of metabolism and mitochondria in biopsies of colorectal tumors as compared to normal and adjacent tissues: bulk transcriptome dataset GSE44076. (**A**): Heatmap of metabolism enrichment performed on tumor and normal controls in the transcriptome (“keggmetascore” R-package); (**B**): differential metabolism enrichment found between CRC tumors and their normal controls; (**C**): principal component analysis performed with metabolism enrichment and stratified on tissue origin; (**D**): principal component analysis performed with metabolism enrichment and stratified by patient gender; (**E**): heatmap of mitochondria enrichment performed on tumor and normal controls in the transcriptome (“mitoscore” R-package); (**F**): differential mitochondrial enrichment found between CRC tumors and their normal controls; (**G**): principal component analysis performed with mitochondria enrichment and stratified on tissue origin; and (**H**): principal component analysis performed with mitochondria enrichment and stratified by patient gender. In subfigures (**B**,**F**), red bullets refer to significantly regulated gene sets, and yellow bullets refer to non-significantly regulated gene sets.

To evaluate metabolism/mitochondria deregulation in tumor samples as compared to normal tissues (adjacent paired normal control and normal mucosa of healthy donors) bulk transcriptome dataset GSE44076 (Table 1) was investigated for quantification with the keggmetascore and mitoscore R-packages. At the metabolism level, a major reduction in metabolism activity was observed in tumor samples as compared to both normal controls (Figure 4A), and this was confirmed by differential analysis (Figure 4B) and by principal component analysis (Figure 4C) without the influence of gender (Figure 4D). Meanwhile, a significant increase in metabolism in colorectal tumors was also observed, affecting alanine, aspartate and glutamate, pyrimidine, purine, and glyoxylate and dicarboxylate metabolism (Figure 4A,B). Concerning mitochondria activities, the majority of them were found to be reduced in tumor samples, with diverse activities implicated in calcium homeostasis ETC, cell death, lipid metabolism, etc. (Figure 4E,F). However, other mitochondria activities were found to be increased in tumor samples, such as the metabolism of mitochondrial nucleic acid protein homeostasis, fission, and cristae formation (Figure 4F). Mitochondria activities effectively stratified tumor samples from their controls, as seen via principal component analysis (Figure 4G), with greater heterogeneity in tumors from men than tumors from women (Figure 4H).

### 3.4. Metabolism/Mitochondria Activities Are Associated with Consensus Molecular Classification During Colorectal Cancer

**Figure 5:**
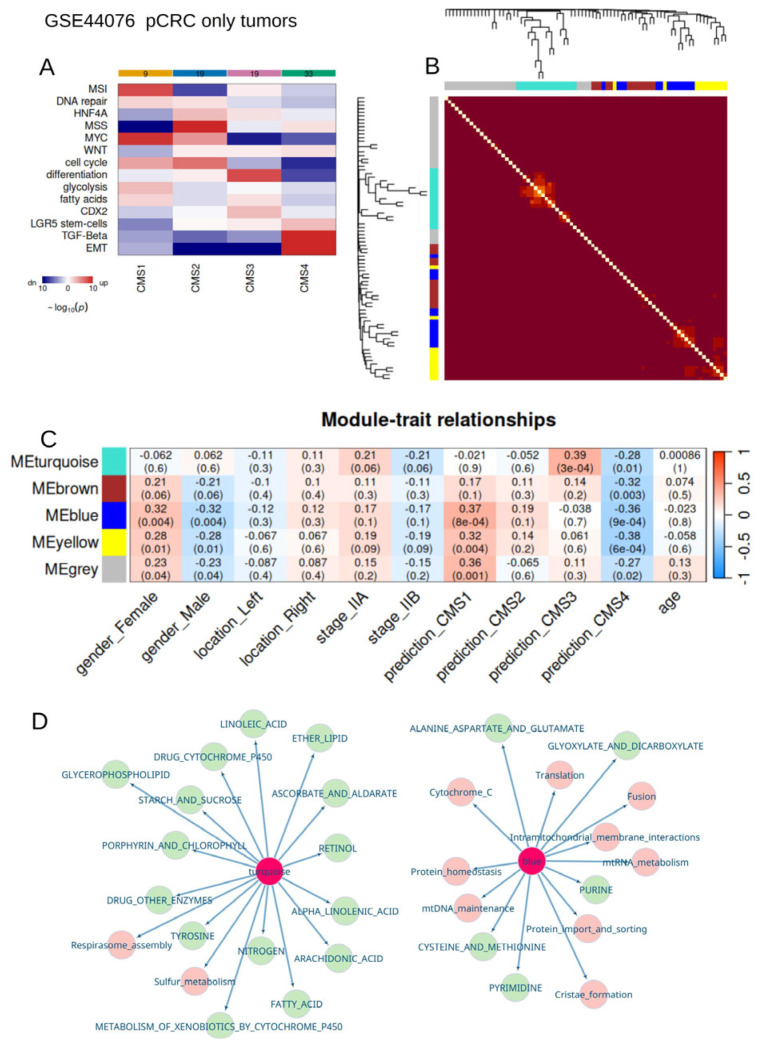
Heterogeneity of mitochondria/metabolism regulations in colorectal tumors according to their consensus molecular subtype classification: only tumor samples from dataset GSE44076. (**A**): Signaling pathway activated in consensus classes from tumors of the GSE44076 dataset; (**B**): heatmap of Topological Overlap Matrix performed during Weighted Gene Co-expression Network Analysis with mitochondria/metabolism enrichment (5 distinct modules); (**C**): heatmap of module/phenotype traits determined via Pearson correlation during WGCNA analysis, performed on mitochondria/metabolism enrichment (Pearson coefficients and Pearson correlation test *p*-value in brackets) (CMS: consensus molecular subtypes); and (**D**): co-enrichment network according to significant modules, mixing mitochondria enrichment (pink) and metabolism enrichment (green).

For tumor transcriptome experiments from dataset GSE44076 (Table 1), consensus molecular classification was predicted with the CMScaller algorithm (Figure 5A and Supplemental Figure S2A) [27]. This analysis predicted 19 CMS2 tumors (canonical proliferative and microsatellite stability), 33 CMS4 tumors (EMT and TGFbeta), 19 CMS3 tumors (metabolic with differentiation), and 9 CMS1 tumors (immune with microsatellite instability and myc—high) (Figure 5A). Based on the mitochondria and metabolism quantification of these tumor samples, a co-expression network analysis by the WGCNA algorithm [28,29] was initiated on this cohort, determining an optimal soft-thresholding power of 22 (Supplemental Figure S2C). Five mitochondria/metabolism modules were characterized by the tumor heterogeneity of this cohort (Figure 5B and Supplemental Figure S2D,E). Phenotype trait correlation analysis highlighted a significant positive correlation between the turquoise module and CMS3-metabolism tumor cluster (Pearson r = 0.39, *p*-value = 3 × 10^−4^, Figure 5C), and the turquoise module was predominantly composed of metabolism gene sets (left turquoise module, Figure 5D). Also, a significant positive correlation was observed between the blue module and the CMS1-MSI immune tumor cluster (Pearson r = 0.37, *p*-value = 8 × 10^−4^, Figure 5C). This blue module was composed of mitochondria and the metabolism geneset (right blue module, Figure 5D) and particularly observed in female patients (Pearson r = 0.32, *p*-value = 0.004, Figure 5C). All five modules were found to be significantly negatively correlated to CMS4 tumors (Figure 5C). These associations were found to be independent of the left/right location of the tumor and of the age of the patients (Figure 5C).

### 3.5. Colorectal Tumors Mutated for BRAFV600E Harbored Strong Metabolism Repression Associated with Fewer Intramitochondrial Membrane Interactions

**Figure 6:**
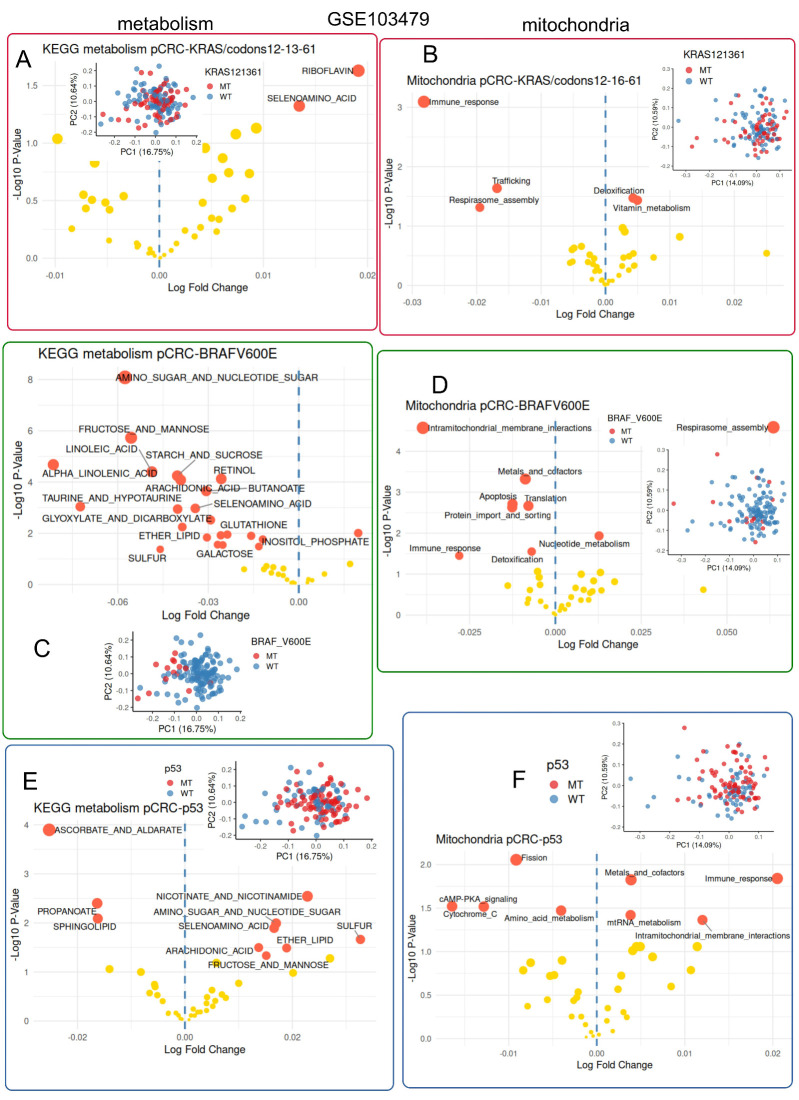
Colorectal tumors mutated for BRAFV600E harbored major metabolism repression: bulk transcriptome dataset GSE103479. (**A**): differential metabolism enrichment analysis between colorectal tumors according to their KRAS mutation status (codons 12, 13, and 61); (**B**): differential mitochondria enrichment analysis between colorectal tumors according to their KRAS mutation status (codons 12, 13, and 61); (**C**): differential metabolism enrichment analysis between colorectal tumors according to their BRAF mutation status (V600E); (**D**): differential mitochondria enrichment analysis between colorectal tumors according to their BRAF mutation status (V600E); (**E**): differential metabolism enrichment analysis between colorectal tumors according to their TP53 mutation status; and (**F**): differential mitochondria enrichment analysis between colorectal tumors according to their TP53 mutation status. For all sub-figures, red bullets refer to significantly regulated gene sets, and yellow bullets refer to non-significantly regulated gene sets.

Colorectal tumors from bulk transcriptome dataset GSE103479 were characterized for the mutation status of TP53, KRAS, and BRAF-V600E (Table 2). Metabolism and mitochondria activities were quantified, respectively, with the keggmetascore and mitoscore R-packages (Supplemental Figure S3A,B). According to the mutational status of KRAS (positions 12, 13, and 61), differential expression and unsupervised principal component analyses were performed on the metabolism scores from tumor samples. These analyses did not show a marked association of metabolism regulation with the KRAS mutation status (Figure 6A). Similar analyses were performed on mitochondria scores, and, except for repression of mitochondrial immune response, no strong association was observed between mitochondria activity and KRAS mutation status in this cohort (Figure 6B). Concerning the mutational status of BRAF-V600E, similar analyses were performed, and strong repression of metabolism activity was observed in BRAF-V600E mutated tumors as compared to wild-type ones (Figure 6C), with the association of some mitochondria activities like intra-mitochondrial membrane interactions, metals and cofactors, apoptosis, translation, protein import and sorting, immune response and detoxification (Figure 6D). Concerning TP53 mutation status, both the repression and activation of certain metabolism pathways could be observed (Figure 6E), as well as both the repression and activation of certain mitochondrial activities (Figure 6F).

### 3.6. Metabolism/Mitochondria Activity Score Is an Independent Parameter to Predict BRAF-V600E Mutation Status During Colorectal Cancer

**Figure 7:**
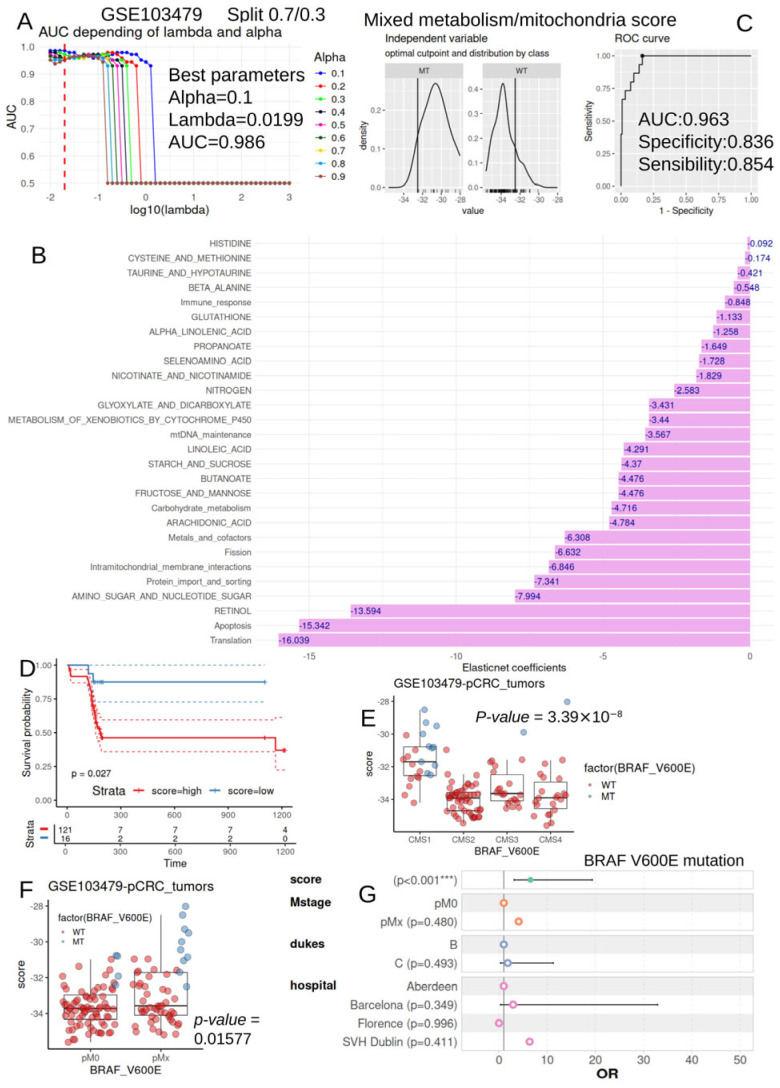
Mitochondria/metabolism enrichment score is associated with the prognosis of colorectal cancer: transcriptome dataset GSE103479. (**A**): Elastic-net tuning on alpha and lambda parameters to predict BRAF-V600E status in colorectal tumors according to metabolism/mitochondria enrichments (cohort split training: 0.7/validation: 0.3); (**B**): barplot of twenty-eight mitochondria/metabolism enrichments with negative Elastic-net coefficients to predict BRAF-V600E mutation status in colorectal tumors; (**C**): optimal threshold determination of the mitochondria/metabolism enrichment score according to the BRAF-V600E mutation status (AUC: area under the curve); (**D**): Kaplan–Meier plot and log rank test analysis for mixed mitochondria/metabolism enrichment score according to the overall survival of the patients; (**E**): boxplot of mixed mitochondria/metabolism enrichment according to CMS consensus classification (Fisher ANOVA *p*-value) and BRAF-V600E mutation status; (**F**): boxplot of mixed mitochondria/metabolism enrichment score according to metastasis stage (2 sided *t* test *p*-value) and BRAF-V600E mutation status; and (**G**): multi-variable logistic model with BRAF-V600E mutation status as the outcome and integrating the mixed mitochondria/metabolism activity score, metastasis stage, Dukes staging, and hospital of sampling as covariates (OR: odds ratios); (***) *p*-value inferior to 0.001,the color of the points is distinct depending on the covariates included in the model.

Metabolism and mitochondria scores (Supplemental Figure S3) were assembled in a common matrix for tumors of the bulk transcriptome dataset GS103479 (Table 2). According to binomial outcomes of BRAF-V600E mutation status, Elastic-net tuning was performed after splitting the cohort into training (0.7) and validation sets (0.3). These learning iterations allowed us to identify the optimal alpha parameter of 0.1 for an area-under-the-curve prediction of 0.99 (Figure 7A). Elastic-net was fit to the best alpha parameter of 0.1 (Supplemental Figure S4A) to select predictive gene sets after cross-validation (Supplemental Figure S4B). Twenty-eight metabolism/mitochondria activities harbored negative predictive coefficients for BRAF-V600E mutation (Figure 7B). According to Elastinet coefficients and quantification of these activities, a metabolism/mitochondria activity score was computed. Optimal threshold determination for this score was determined according to BRAF-V600E mutation (Figure 7C). This score predicted the BRAF-V600E mutation status with an area under the curve of 0.96, a specificity of 0.84, and a sensibility of 0.85. Patients with high score values had a greater risk to metastasis (*p*-value = 0.0058267, Table 2), and, according to consensus molecular classification, they comprised a high proportion in the immune-CMS1 (MSI) patient group (*p*-value < 0.001, Table 2). Patients presenting high values of the metabolism/mitochondria activity score were predicted to have a significant worse prognosis by univariate overall survival analysis (log rank test *p*-value = 0.027, Figure 7D). According to consensus molecular classification, they comprised a high proportion in the immune-CMS1 (MSI) patient group (*p*-value < 0.001, Table 2). This last observation was confirmed by analysis of variance (Fisher one WAY ANOVA *p*-value = 3.39 × 10^−8^, Figure 7E). Patients with high score values had a greater risk to metastasis (*p*-value = 0.0058267, Table 2), confirmed by a two-sided Student test (*p*-value = 0.015, Figure 7F). A logistic multi-variable model was built with BRAF-V600E mutation status as the binary outcome, including relevant epidemic/clinical parameters such as the collecting center, Duke stage, metastasis status, and computed metabolism/mitochondria activity score (Figure 7G). The metabolism/mitochondria activity score was confirmed as an independent positive parameter to predict the BRAF-V600E mutation status of colorectal tumors (multi-variable model: odds ratios: 6.53 and *p*-value = 3.32 × 10^−5^, Table 3).

A nomogram of the multi-variable model confirmed the importance of the metabolism/mitochondria activity score in the total amount of points in the model (Supplemental Figure S4C), independent of the variance observed in the Florence hospital, which only harbored patients with the wild-type mutation BRAF-V600E (Supplemental Figure S4D).

## 4. Discussion

During this work, two R-packages dedicated to quantifying metabolism (based on KEGG metabolism pathways) and mitochondria (based on mitocarta V3 annotations) activities in transcriptome data were designed.

Applying the use of both the “mitoscore” and “keggmetascore” R-packages to the study of colorectal cancer, in LOVO cells we functionally confirmed both regulation of metabolism and mitochondria activities after stimulation with metformin. Metformin, a commonly prescribed drug for type 2 diabetes, has been extensively studied for its potential anti-cancer effects, including in colorectal cancer (CRC). Experimental evidence suggests that the mechanisms underlying the suppression on aberrant crypt foci formation of metformin are associated with the inhibition of mTOR resulting from the activation of AMPK [38,39,40]. The anti-diabetic drug metformin is recognized as a dual AMPK activator and ETC complex I inhibitor at the mitochondria level [41,42]. In cancer, it acts synergistically with chemotherapy to eradicate resistant tumor cells and promote cancer remission [43,44,45]. Early stimulation of LOVO cells with metformin (8 h) showed strong repression of cAMP-PKA in mitochondrial activity. Cyclic AMP-dependent protein kinase is able to phosphorylate Drp1, an important mitochondrial actor implicated in mitochondrial fragmentation and recognized as an early event in apoptosis [46]. It is also known that the GPR176/GNAS complex inhibits mitophagy via the cAMP/PKA/BNIP3L axis, thereby promoting the tumorigenesis and progression of CRC [47]. By contrast, at 24 h of stimulation, metformin stimulation was found to have upregulated mitochondrial activity through cristae formation, cytochrome C, calcium uniporter and homeostasis, detoxification, and complex II when amino acid (Taurine, beta alanine, phenylalanine) and lipid (ether lipid, alpha linolenic acid, or linolenic acid) metabolism were also increased. Concerning lipid metabolism, a strong expression of fatty acid synthase (FASN) and ATP-citrate lyase (ACYL) is known to occur during colon cancer [48,49], and their inhibition was found to impair metastatic progression in colon cancer cells by reducing CD44 and hepatocyte growth factor receptor-mediated signaling, resulting in reduced tumor cell migration and in vitro clonogenicity [50,51,52].

In the last decade, immune checkpoint inhibitors (ICIs) have revolutionized cancer treatment and have rejuvenated the field of tumor immunology. At the single-cell level, in single-cell RNA sequencing of colorectal tumors we observed the joint activation of metabolism and mitochondria activities in tumor cells from MSI-high tumors; by contrast, joint repression of metabolism and mitochondria activities in tumor cells from POLE mutated tumors was observed. These two types of tumors are known to have distinct responses to immune checkpoint blockage therapy. Studies evaluating immune checkpoint inhibitors in colorectal cancer failed to demonstrate their benefit, with the exception of patients with MSI-tumors [12,53]. Therefore, the opposite metabolism and mitochondria activity between MSI-high tumors and POLE mutated tumors could provide a new angle to identify new molecular targets and explain their different responses to ICI therapy.

A metabolomic study performed on colorectal tumors revealed metabolites consistent with enhanced glycolysis and glutaminolysis [54,55,56]. Another study performed by gas chromatography and mass spectrometry revealed 19 metabolites deregulated between the colorectal tumor and adjacent mucosa [57]. The limitation of these studies is that they characterized colorectal cancer metabolism without taking inter-patient heterogeneity into account. At the bulk transcriptome level, colorectal tumors present fewer metabolism/mitochondria activities as compared to normal tissues—adjacent tissue and normal mucosa. Multi-modal integration by co-expression network analysis has shown that metabolism/mitochondria activities are associated with consensus molecular subtype classification of colorectal cancer. Consensus molecular subtype classification (CMS) identified four subtypes of colorectal cancer with distinct transcriptomic patterns that could potentially be translated into different prognosis responses [58,59].

Oxidative phosphorylation (OXPHOS) occurs at the mitochondria electron transport chain (ETC) and is a highly efficient pathway for ATP production in the cell [60,61]. Complete abolition of ETC activity was shown to prevent tumorigenesis in a Kristen Rat Sarcoma (KRAS)-driven mouse model of lung cancer [62]. According to the KRAS, BRAF, and TP53 driver gene mutation status, our analysis based on both “mitoscore” and “keggmetascore” R-packages revealed strong repression of metabolism pathways, which was mainly associated with fewer intra-mitochondrial membrane interactions in tumors harboring the BRAF-V600E mutation. Machine learning Elastic-net allowed us to build a mixed metabolism/mitochondria activity score, which was found to be an independent parameter predictive of BRAF-V600E mutation status in colorectal cancer. In colorectal cancer, BRAF mutations also predict failure of the epidermal growth factor receptor inhibitors cetuximab and panitumumab and are associated with shorter overall and progression-free survival [63,64]. A high metabolism/mitochondria activity score predicting BRAF-V600E status was particularly associated with patients presenting metastasis. Mitochondria dysfunction can favor pro-metastatic behavior either by promoting glycolytic compensation [65,66] or by impacting the NAD+/NADH redox ratio that regulates Sirtuin activity, thereby directly promoting tumor metastasis [67]. High oxidative phosphorylation (OXPHOS) activity could induce stronger resistance to apoptosis through the induction of a pro-oxidant state [68,69,70]. Dysregulated mitochondrial metabolism was associated with metastasis promotion by inducing stress tolerance during nutriment deprivation [67,68,69,70,71]. Mutations affecting the BRAF gene were present in approximately 8–12% of metastatic colorectal cancer; BRAF is a key driver gene of mitogen-activated protein kinase (MAPK) activation, and its mutations are associated with a poorer response to standard treatments and worse prognosis [72,73]. BRAF-V600E-mutated metastatic colorectal cancer represents a distinct clinical and pathological entity compared to wild-type colorectal cancer, defining a specific subgroup. These tumors are more frequently observed in females, with the tumors being right-sided, mucinous, or poorly differentiated [74].

## 5. Conclusions

During this work, we highlighted some major biological findings, included as follows: 1. the association of metabolism/mitochondria activities with consensus molecular subtype (CMS) classification, particularly CMS1 (immune/MSI) and CMS3 (metabolic); 2. strong repression of metabolism pathways associated with the BRAF-V600E mutation status; 3. the development of the MM_score, which is an independent parameter predictive of BRAF-V600E mutation status and associated with worse prognosis and metastatic samples; and 4. the discovery of opposing metabolic/mitochondrial activity in tumor cells from MSI-high vs. POLE-mutated tumors, directly relevant to their distinct responses to immune checkpoint blockade (ICI) therapy.

## Figures and Tables

**Table 1:** Clinical and epidemiological parameters of tissues processed in transcriptome cohort GSE44076. This table summarized clinical and epidemiological parameters of 50 colon mucosa from healthy donor samples as compared to 98 paired samples from colorectal cancer patients (normal: normal adjacent tissue; tumor: malignant tissue). Quantitative variables were tested by ANOVA (mean (sd: standard deviation)), and qualitative variables were tested by chi square test (number (percentage)). Significance was assessed with a *p*-value ≤ 0.05.

Variable	Level	Mucosa (*n* = 50)	Normal (*n* = 98)	Tumor (*n* = 98)	Total (*n* = 246)	*p*-Value
Age at diagnosis	Mean (sd)	62.5 (14.2)	70.5 (9)	70.5 (9)	68.9 (10.7)	<1 × 10^−4^
Gender	Male	27 (54.0)	71 (72.4)	71 (72.4)	169 (68.7)	
	Female	23 (46.0)	27 (27.6)	27 (27.6)	77 (31.3)	0.04273
Tumor location	Left	23 (46.0)	60 (61.2)	60 (61.2)	143 (58.1)	
	Right	27 (54.0)	38 (38.8)	38 (38.8)	103 (41.9)	0.15003
Tumor stage	IIA	not available	not available	90 (91.8)	90 (91.8)	
	IIB	not available	not available	8 (8.2)	8 (8.2)	not available

**Stage IIA** means the tumor has grown through the muscular wall of the colon/rectum into the outermost layers but has not spread to nearby lymph nodes or distant organs. **Stage IIB** indicates the tumor has penetrated even further, reaching or invading nearby tissues beyond the colon wall, still without lymph node or distant metastasis

**Table 2:** Clinical and epidemiological parameters of tissues processed in the transcriptome cohort GSE103479. Column low: samples with a low mitochondria/metabolism score; column high: samples with a high mitochondria/metabolism score; and column total: description of the total samples from the cohort. Quantitative variables were tested by Student’s *t*-test (mean (sd: standard deviation)), and qualitative variables were tested by the chi square test (number (percentage)). Significance was assessed with a *p*-value ≤ 0.05.

Variable	Level	Low (*n* = 102)	High (*n* = 35)	Total (*n* = 137)	*p*-Value
Hospital	Aberdeen	10 (9.8)	14 (40.0)	24 (17.5)	
	Barcelona	41 (40.2)	11 (31.4)	52 (38.0)	
	Florence	17 (16.7)	0 (0.0)	17 (12.4)	
	SVH Dublin	34 (33.3)	10 (28.6)	44 (32.1)	0.0001612
tumor_size	mean (sd)	4.9 (1.8)	5.6 (2.6)	5.1 (2.1)	0.1030527
Tumor stage	pT4	3 (2.9)	6 (17.1)	9 (6.6)	
	pT3	77 (75.5)	22 (62.9)	99 (72.3)	
	pT2	5 (4.9)	1 (2.9)	6 (4.4)	
	pT4a	8 (7.8)	2 (5.7)	10 (7.3)	
	pT4b	8 (7.8)	4 (11.4)	12 (8.8)	
	pT1	1 (1.0)	0 (0.0)	1 (0.7)	0.0838208
Nodule stage	pN0	57 (55.9)	17 (48.6)	74 (54.0)	
	pN1	20 (19.6)	5 (14.3)	25 (18.2)	
	pN2	5 (4.9)	3 (8.6)	8 (5.8)	
	pN2a	3 (2.9)	2 (5.7)	5 (3.6)	
	pN1a	8 (7.8)	4 (11.4)	12 (8.8)	
	pN1b	6 (5.9)	2 (5.7)	8 (5.8)	
	pN2b	3 (2.9)	2 (5.7)	5 (3.6)	0.8400949
Metastasis stage	pMx	35 (34.3)	22 (62.9)	57 (41.6)	
	pM0	67 (65.7)	13 (37.1)	80 (58.4)	0.0058267
lymph_nodes_excised	mean (sd)	21.8 (16)	18.8 (8.7)	21 (14.5)	0.2857346
age_diagnosis	mean (sd)	69.9 (11.3)	70.4 (12.3)	70 (11.5)	0.8049928
CMS	CMS1	6 (6.7)	17 (58.6)	23 (19.3)	
	CMS2	50 (55.6)	2 (6.9)	52 (43.7)	
	CMS4	18 (20.0)	4 (13.8)	22 (18.5)	
	CMS3	16 (17.8)	6 (20.7)	22 (18.5)	< 1 × 10^−4^
BRAF_V600E mutation	WT	102 (100.0)	20 (57.1)	122 (89.1)	
	MT	0 (0.0)	15 (42.9)	15 (10.9)	< 1 × 10^−4^
TP53 mutation	WT	40 (39.2)	19 (54.3)	59 (43.1)	
	MT	62 (60.8)	16 (45.7)	78 (56.9)	0.1751699
KRAS (12-13-61) position mutations	MT	42 (41.2)	12 (34.3)	54 (39.4)	
	WT	60 (58.8)	23 (65.7)	83 (60.6)	0.603493
Overall Survival (months)	mean (sd)	233.2 (292.9)	146.2 (47.4)	211 (256.4)	0.0810742
Overall survival status	alive	60 (58.8)	22 (62.9)	82 (59.9)	
	dead	42 (41.2)	13 (37.1)	55 (40.1)	0.8256887
Progression free survival (months)	mean (sd)	55.5 (41.2)	44.1 (32.4)	52.6 (39.4)	0.138873
Recurrence status	no	73 (73.0)	24 (68.6)	97 (71.9)	
	yes	27 (27.0)	11 (31.4)	38 (28.1)	0.7771385
Adjuvant therapy	yes	42 (42.4)	13 (37.1)	55 (41.0)	
	no	57 (57.6)	22 (62.9)	79 (59.0)	0.7292902

**Table 3:** Multivariable logistic model to predict BRAF-V600E mutation status in colorectal tumors: (*): *p*-value < 0.05.

Term	Odds Ratios	CI95-Low	CI95-High	*p* Value
Metabolism/mitochondria score	6.53 × 10^0^	3.13 × 10^0^	1.94 × 10^1^	3.32 × 10^−5^ *
Mstage (pMx)	4.09 × 10^0^	1.51 × 10^−1^	3.17 × 10^2^	4.80 × 10^−1^
Dukes (Stage C)	1.82 × 10^0^	3.33 × 10^−1^	1.13 × 10^1^	4.93 × 10^−1^
Hospital (Barcelona)	2.93 × 10^0^	3.24 × 10^−1^	3.29 × 10^1^	3.49 × 10^−1^
hospital (Florence)	3.27 × 10^−6^	non available	3.44 × 10^77^	9.96 × 10^−1^
Hospital (SVH-Dublin)	6.36 × 10^0^	1.43 × 10^−1^	9.00 × 10^2^	4.11 × 10^−1^

pMx: pathological assessment of metastasis; SVH: **St. Vincent’s Hospital**.

## Data Availability

The data presented in this study are openly available. These data were derived from the following resources available in the public domain: Jupyter notebook for processing mitoscore R-package quantification starting from a Seurat 5 single cell-RNAseq R object: https://github.com/cdesterke/CRC_metamito/blob/main/mitochondria.ipynb (accessed on 23 April 2025). keggmetascore R-package install repository: https://github.com/cdesterke/keggmetascore (accessed on 22 April 2025). mitoscore R-package install repository: https://github.com/cdesterke/mitoscore (accessed on 22 April 2025).

## Supplementary Materials

The following supporting information can be downloaded at https://www.mdpi.com/article/10.3390/jpm15120571/s1, Supplemental Figure S1: single-cell FeaturePlot for expression of tumor markers in cluster number “3” from GSE222300 sc-RNAseq; Supplemental Figure S2: heterogeneity of metabolism/mitochondrial activities in tumors of colorectal cancer according to consensus molecular subtype classification; Supplemental Figure S3: metabolism and mitochondria quantification in tumors from transcriptome dataset GSE103479; Supplemental Figure S4: independence of the metabolism/mitochondria score to predict BRAF-V600E mutation status in colorectal tumors.

## Abbreviations

The following abbreviations are used in this manuscript:

ANOVA	Analysis of variance
CMS	Consensus molecular subtypes
CRC	Colorectal cancer
OXPHOS	Oxidative phosphorylation
