# Supplementary material for: The Heterogeneous Interplay Between Metabolism and Mitochondrial Activity in Colorectal Cancer
Source: J Pers Med. 2025 Nov 28;15(12):571. doi: 10.3390/jpm15120571 (PMC12734392; doi:10.3390/jpm15120571)
Supplement: Supplementary file 1 [file jpm-15-00571-s001.zip › jpm-3958683-supplementary.pdf]

Article

Heterogeneous interplay between metabolism and mitochondria activities during colorectal cancer

Christophe Desterke [1], Yuanji Fu [2], Jorge Mata-Garrido [3], Ahmed Hamaï [4] and Yunhua Chang [2,*]

Supplemental Figures

Supplemental Figure S1. Single cell FeaturePlot for expression of tumor markers in cluster number "3" from GSE222300 sc-RNAseq

Supplemental Figure S2. Heterogeneity of metabolism/mitochondrial activities in tumor of colorectal cancer according consensus molecular subtype classification

Supplemental Figure S3. Metabolism and mitochondria quantification in tumors from transcriptome dataset GSE103479

Supplemental Figure S4. independence of the metabolism/mitochondria score to predict BRAF-V600E mutation status in colorectal tumors

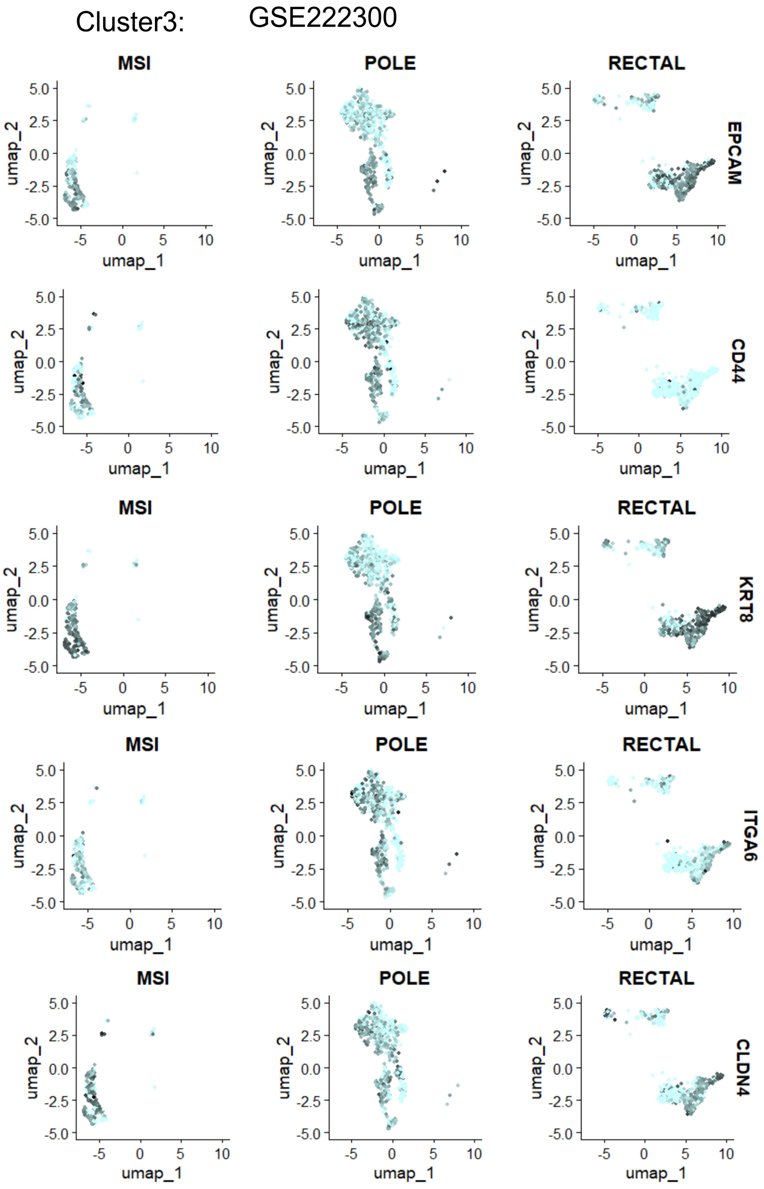

Supplemental Figure S1. Single cell FeaturePlot for expression of tumor markers in cluster number "3" from GSE222300 sc-RNAseq: : EPCAM, CD44, KRT8, ITGA6, and CLDN4.

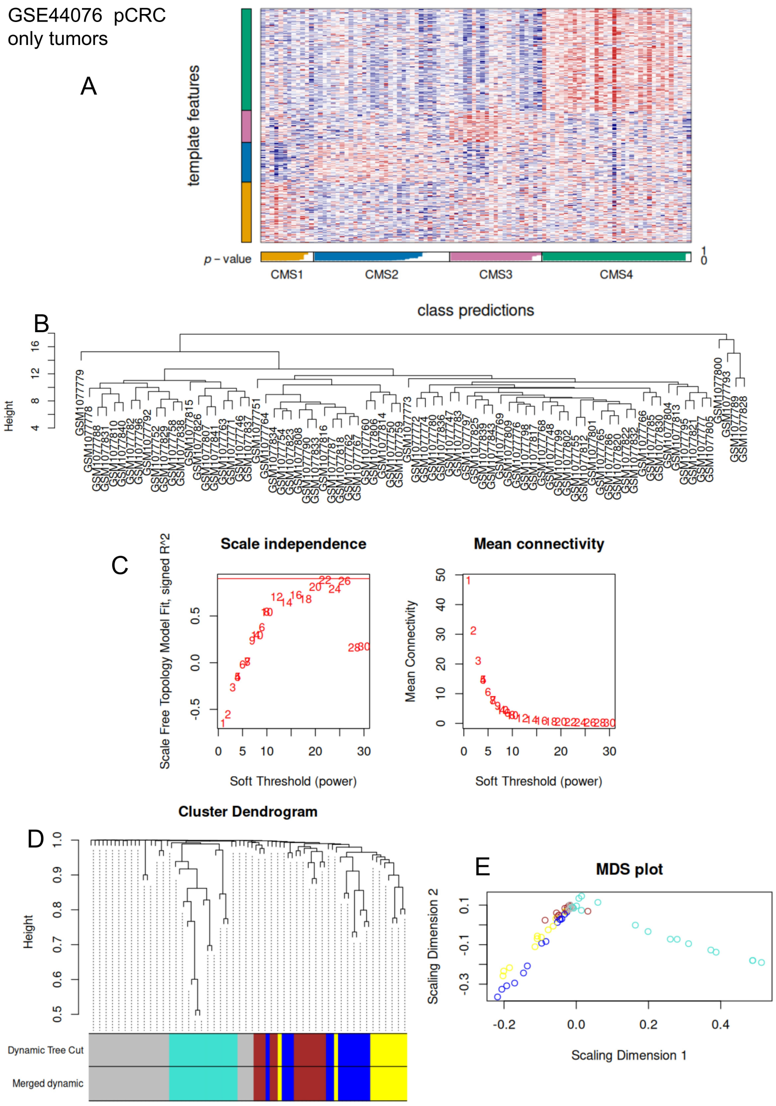

Supplemental Figure S2. Heterogeneity of metabolism/mitochondrial activities in tumor of colorectal cancer according consensus molecular subtype classification: A/ Prediction of the consensus molecular subtypes (CMS) for tumor of the bulk transcriptome dataset GSE44076; B/ Hierarchical clustering of the tumor samples from GSE44076 transcriptome dataset according metabolism/mitochondria activities scored receptively quantified by “keggmetascore” and “mitoscore” R-packages; C/ Tuning of the self power for WGCNA co-expression network analysis based on metabolism/mitochondria activities in tumor transcriptome from GSE44076 dataset; D/ Gene set module detection during WGCNA analysis based on metabolism/mitochondria activities in tumor transcriptome from GSE44076 dataset; E/ Multi-dimension scaling dimension reduction performed on metabolism/mitochondria modules detected in tumors of GSE44076 transcriptome dataset.

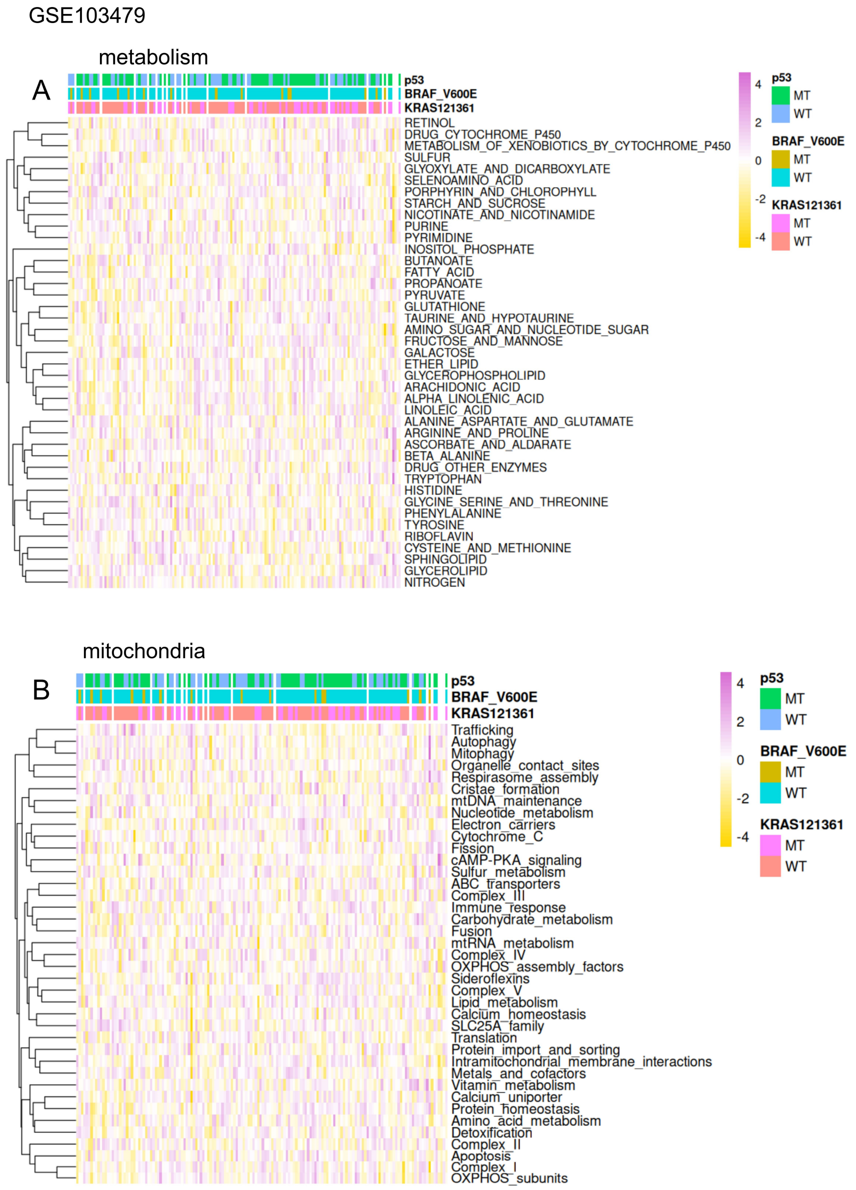

Supplemental Figure S3. Metabolism and mitochondria quantification in tumors from transcriptome dataset GSE103479: A/ Heatmap of metabolism scores obtained with "keggmetascore" R-package on tumors from transcriptome dataset GSE103479; B/ Heatmap of mitochondria scores obtained with "mitoscore" R-package on tumors from transcriptome dataset GSE103479.

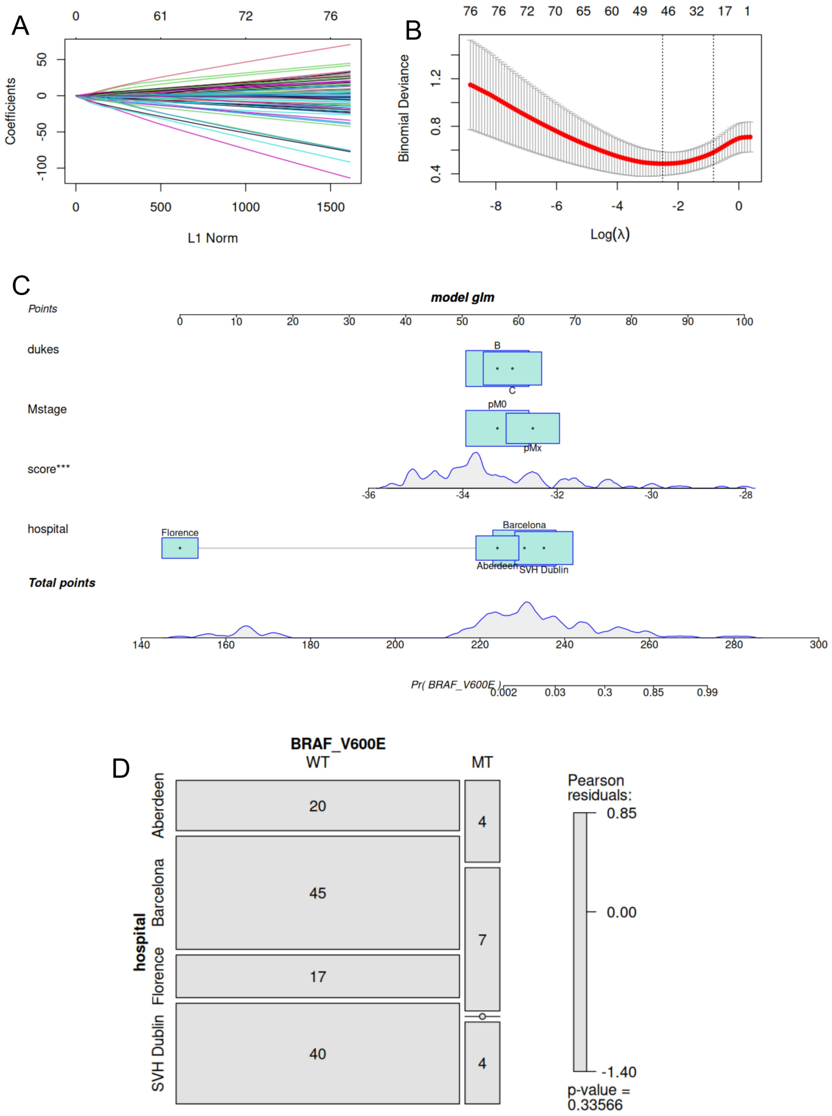

Supplemental Figure S4. independence of the metabolism/mitochondria score to predict BRAF-V600E mutation status in colorectal tumors: A/ Fit of the Elastic-net model based on metabolism/mitochondria activities to predict binomial status of the BRAF-V600E mutation status with alpha parameter fixed to 0.1; B/ Cross-validation fitting to select predictive gene sets for a range of lambda parameter and a best alpha parameter fixed to 0.1; C/ Nomogram of multi-variable logistic model for prediction of the BRAF-V600E mutation status as binary outcome; D/ MosaicPlot testing association between recruitment center and BRAF-V600E mutation status.
